# Clinical Characteristics, Etiologies, and Management Outcomes of Antenatal Hydronephrosis: A Prospective Observational Study

**DOI:** 10.7759/cureus.97570

**Published:** 2025-11-23

**Authors:** Harsha Vardhan R Nidanavada, Hemanth S Ghalige, Arunkumar Tukaram, Nandhinipriya

**Affiliations:** 1 General Surgery, Employees' State Insurance Corporation Medical College (ESIC-MC) &amp; Post Graduate Institute of Medical Sciences and Research (PGIMSR), Bengaluru, IND; 2 Surgery, Employees' State Insurance Corporation Medical College (ESIC-MC) &amp; Post Graduate Institute of Medical Sciences and Research (PGIMSR), Bengaluru, IND

**Keywords:** antenatal hydronephrosis, hydronephrosis severity, postnatal evaluation, prenatal ultrasonography, urological anomalies

## Abstract

Background

Hydronephrosis detected during prenatal imaging is among the more frequently encountered fetal renal findings. Although many cases resolve without intervention, some may signify underlying structural abnormalities of the urinary tract that warrant closer evaluation and possible treatment. This study aimed to assess the clinical patterns, causes, and outcomes of postnatal management in neonates and infants diagnosed prenatally with hydronephrosis.

Methods

A prospective observational study was conducted involving 50 neonates and infants with prenatal evidence of hydronephrosis. Initial postnatal ultrasonography was performed within the first few weeks of life, and additional investigations - such as micturating cystourethrograms and radionuclide scans - were undertaken selectively. Parameters recorded included gender, gestational age at detection, laterality, severity based on ultrasonographic grading, final diagnosis, and management approach. Hydronephrosis was categorized as mild, moderate, or severe. Statistical analysis was used to evaluate the association between the severity and the chosen treatment pathway.

Results

There was a higher number of male infants in the study group. Most cases were identified during mid-trimester anomaly scans. Involvement was equally distributed between right-sided and bilateral hydronephrosis, with fewer left-sided cases. Over half of the cases were mild, with fewer instances classified as moderate or severe. The most frequent underlying conditions included obstructive and reflux-related pathologies. Conservative observation proved effective in the majority of cases, especially in those with less severe involvement, whereas a minority with severe hydronephrosis required surgical correction. A significant correlation was noted between the degree of hydronephrosis and the need for operative intervention.

Conclusion

Prenatally identified hydronephrosis includes a range of potential causes that require a structured postnatal diagnostic approach. The severity seen on early imaging is a reliable indicator of likely management needs. Timely evaluation and appropriate monitoring enable effective differentiation between cases that will self-resolve and those requiring surgical care, supporting both clinical decision-making and parental guidance.

## Introduction

Antenatal hydronephrosis (ANH) is one of the most frequently identified fetal anomalies, with detection rates rising due to the widespread use of prenatal ultrasonography [1]. Occurring in approximately 1-5% of pregnancies, ANH presents both diagnostic and management challenges, particularly in distinguishing physiological dilatation from pathological causes [2]. Although many cases resolve spontaneously after birth, a subset of neonates may have significant underlying congenital anomalies of the kidney and urinary tract (CAKUT), necessitating structured postnatal evaluation and, in some instances, surgical intervention [3]. The diagnosis of ANH is based on prenatal sonographic detection of renal pelvic dilatation, with the anteroposterior diameter (APD) of the renal pelvis serving as a key metric to assess severity. Generally, an anteroposterior renal pelvic diameter (APD) greater than 4 mm in the second trimester and greater than 7 mm in the third trimester is considered abnormal. As per the Society for Fetal Urology (SFU) guidelines, antenatal hydronephrosis (ANH) is typically classified into mild (4-<7 mm), moderate (7-10 mm), and severe (>10 mm) categories, with increasing APD measurements corresponding to a higher risk of postnatal uropathies [3,4]. Despite routine anomaly scans in the second trimester identifying ANH with relative ease, the absence of standardized postnatal follow-up - particularly in rural or low-resource settings - can lead to delays in diagnosing clinically significant urological conditions [5]. The underlying pathophysiology often involves impaired fetal urinary drainage, which may result from mechanical obstruction at various levels of the urinary tract or from transient, physiological factors. Fetal urine production begins as early as 10-12 weeks of gestation, and any obstruction to urinary outflow can cause progressive renal pelvic dilatation, potentially affecting renal structure and function [6]. Obstructive causes of ANH include pelviureteric junction obstruction (PUJO), posterior urethral valves (PUV), vesicoureteral reflux (VUR), and ureteroceles. Non-obstructive causes, such as physiological or transient hydronephrosis, often resolve without intervention in the postnatal period [7]. The clinical relevance of ANH depends largely on the underlying etiology and the degree of dilatation. Moderate to severe ANH is more likely to be associated with adverse outcomes, including recurrent urinary tract infections (UTIs), renal impairment, and chronic kidney disease in severe cases [8]. In contrast, most mild cases resolve spontaneously without long-term sequelae [8,9]. Early identification of high-risk cases is essential to guide appropriate intervention while avoiding unnecessary procedures in low-risk infants. Several postnatal factors have been proposed to aid in risk stratification and management, including renal pelvic APD, Society for Fetal Urology (SFU) grading, presence of ureteric dilatation, differential renal function (DRF) on nuclear scans, and drainage patterns indicative of obstruction [2]. Integrating prenatal imaging findings with postnatal functional assessments allows for more accurate prognostication and individualized care. Given the heterogeneity in presentation and outcomes, there is a continued need for clearer criteria to predict which infants will require surgical treatment. This study aims to evaluate antenatally diagnosed hydronephrosis in neonates and infants, identify the underlying etiologies, and explore the relationship between APD measurements and the need for surgical management. By correlating prenatal ultrasound findings with postnatal outcomes, this study seeks to contribute to the development of more standardised and evidence-based management protocols for ANH. This study was undertaken to characterize the clinical features, determine the underlying causes, and evaluate postnatal outcomes of antenatally detected hydronephrosis, with emphasis on correlating severity with management strategies.

## Materials and methods

Study design

This descriptive observational study was conducted at ESIC-MC & PGIMSR, Rajajinagar, from May 2023 to October 2024. The study was approved by the ethics committee of the hospital.

Patients

Convenience sampling was used to recruit participants. Inclusion criteria were neonates with antenatal ultrasonography performed after 20 weeks of gestation showing an anteroposterior renal pelvic diameter (APD) >4 mm (as per Society of Fetal Urology guidelines 2010). Exclusion criteria included infants whose parents or guardians did not provide informed written consent and those diagnosed with hydronephrosis only postnatally.

Imaging and follow-up

Following informed parental consent, clinical history and relevant prenatal investigation data were collected for all neonates with antenatal ultrasonography indicating hydronephrosis. Postnatal renal ultrasound was performed at 3 days, 1 month, and 6 months of life. All infants were followed for a minimum of six months to assess for spontaneous resolution or the development of significant urinary tract pathology. Additional investigations, such as micturating cystourethrogram (MCU) or renal scintigraphy, were performed selectively based on clinical and imaging findings. The criteria and timing for intervention were determined according to multidisciplinary guidelines, involving consensus between pediatric surgery, radiology, and nephrology teams.

Data was entered and processed using Microsoft Excel 2019 (Microsoft Corporation, Redmond, WA, USA). The institution licensed IBM SPSS Statistics for Windows, Version 26.0 (IBM Corp., Armonk, NY, USA) for statistical analysis.

## Results

**Figure 1:**
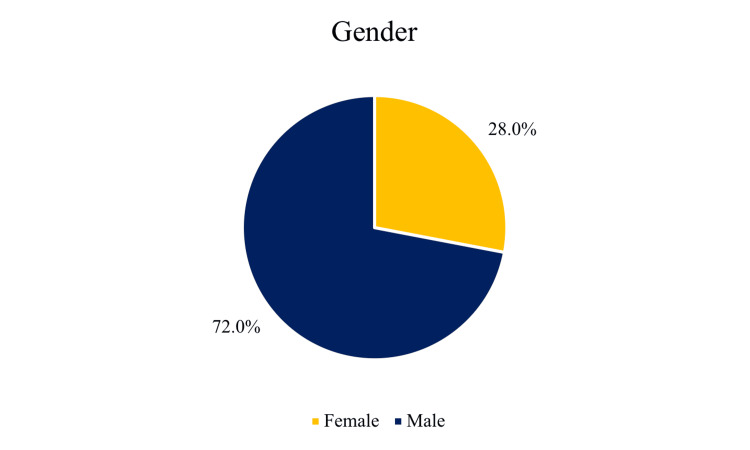
Gender Distribution of Study Population

Our study revealed a clear male predominance among infants with antenatal hydronephrosis, with 36 (72.0%) of participants being male compared to 14 (28.0%) of female participants, as depicted in Figure 1. This notable gender disparity aligns with existing literature suggesting that urological anomalies, including hydronephrosis, occur more frequently in male fetuses. The approximately 2.5:1 male-to-female ratio observed in our cohort may reflect underlying anatomical differences in urinary tract development between genders.

Diagnosis of antenatal hydronephrosis occurred predominantly during the early second trimester, with 26 (52%) of participants identified between 20-24 weeks of gestation, coinciding with routine anomaly scanning. A further 10 (20%) of participants were diagnosed between 25-28 weeks, while only 3 (6%) were identified between 29-32 weeks. Later diagnoses occurred in 6 (12%) of participants at 33-36 weeks and 5 (10%) after 37 weeks, as depicted in Table 1. This distribution highlights the importance of the mid-pregnancy anomaly scan in detecting urinary tract abnormalities, enabling timely post-natal follow-up planning.

**Table 1:** Gestational Age at Diagnosis of Antenatal Hydronephrosis Values are expressed as frequency and percentage.

Gestational Age (weeks)	N	%
20-24	26	52%
25-28	10	20%
29-32	3	6%
33-36	6	12%
>37	5	10%

Laterality analysis revealed an equal distribution between right-sided and bilateral hydronephrosis, each accounting for 18 (36.0%) participants, while left-sided involvement was observed in 14 (28.0%), as depicted in Table 2. The substantial proportion of bilateral cases (over one-third of participants) is clinically significant, as bilateral involvement has been associated with an increased likelihood of underlying pathology requiring intervention compared to unilateral cases.

**Table 2:** Laterality of Antenatal Hydronephrosis Values are expressed as frequency and percentage.

Laterality of Antenatal hydronephrosis	N	%
Bilateral	18	36.0%
Left	14	28.0%
Right	18	36.0%

Classification by severity demonstrated that mild hydronephrosis was the predominant presentation, affecting 27 (54.0%) participants, followed by moderate hydronephrosis in 15 (30.0%) participants, with severe cases comprising only 8 (16.0%), as depicted in Table 3.

**Table 3:** Severity Classification of Antenatal Hydronephrosis Values are expressed as frequency and percentage.

Hydronephrosis severity	N	%
Mild	27	54.0%
Moderate	15	30.0%
Severe	8	16.0%

All participants (100%) underwent post-natal ultrasonography as the initial investigation. More specialized imaging with a radionuclide scan was performed in 22 (44.0%) participants, while micturating cystourethrogram (MCU) was conducted in 24 (48.0%), as depicted in Table 4.

**Table 4:** Post-natal Investigations Performed Values are expressed as frequency and percentage. MCU: Micturating cystourethrogram; USG: Ultrasonography

Post-natal Investigations	N	%
USG	50	100%
Radionuclide Scan	22	44.0%
MCU	24	48.0%

**Figure 2:**
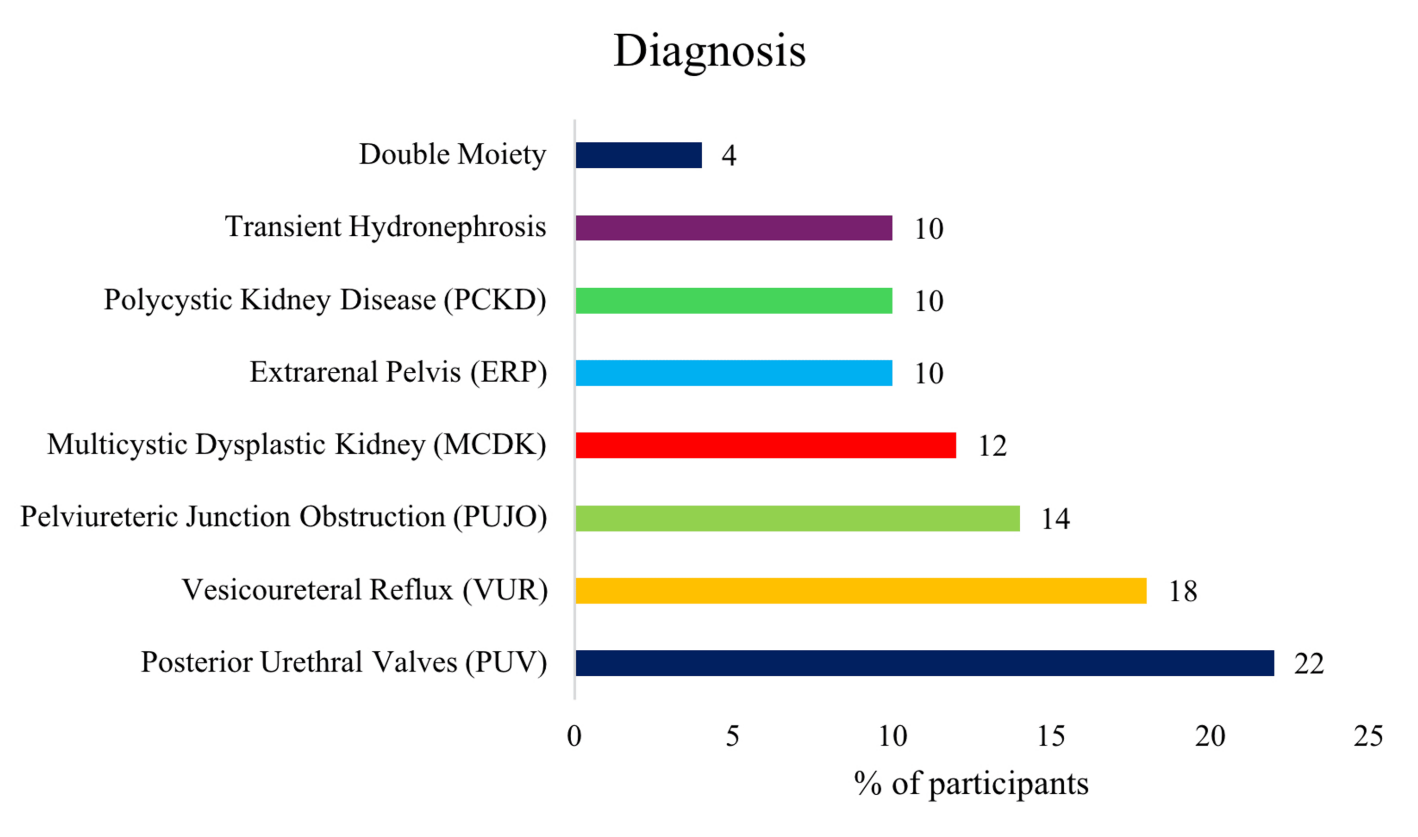
Post-natal Diagnoses in Infants with Antenatal Hydronephrosis

The etiological spectrum revealed Posterior Urethral Valves (PUV) as the most common definitive diagnosis, accounting for 11 (22%) participants, followed by Vesicoureteral Reflux (VUR) in 9 (18%) and Pelviureteric Junction Obstruction (PUJO) in 7 (14%) participants. Multicystic Dysplastic Kidney (MCDK) was diagnosed in 6 (12%) of participants, while Extrarenal Pelvis (ERP), Polycystic Kidney Disease (PCKD), and Transient Hydronephrosis each represented 5 (10%) participants. Double Moiety was the least common diagnosis at 2 (4%) participants, as depicted in Figure 2. This diverse distribution emphasizes the heterogeneous nature of pathologies underlying antenatal hydronephrosis.

Analysis of severity patterns across diagnoses revealed notable variations. PUV cases predominantly presented with moderate hydronephrosis (6 (54.5%) of PUV participants), while most other conditions presented primarily with mild hydronephrosis. VUR, PUJO, MCDK, ERP, PCKD, and Transient Hydronephrosis all showed mild presentations in the majority of cases (5 (55.6%), 4 (57.1%), 4 (66.7%), 3 (60.0%), 3 (60.0%), and 3 (60.0%) of their respective participants) as depicted in Table 5. Notably, MCDK and Transient Hydronephrosis displayed bimodal distributions with significant proportions presenting with either mild or severe hydronephrosis. These severity patterns across diagnoses may serve as valuable clinical indicators for diagnostic suspicion. The analysis reveals no statistically significant association between specific diagnoses and the severity pattern of hydronephrosis (p > 0.05).

**Table 5:** Correlation Between Post-natal Diagnosis and Severity of Hydronephrosis Values are expressed as frequency and percentage. The p-value is by chi-square test, and <0.05 is considered statistically significant. PUV: Posterior Urethral Valves; VUR: Vesicoureteral Reflux; PUJO: Pelviureteric Junction Obstruction; MCDK: Multicystic Dysplastic Kidney; ERP: Extrarenal Pelvis; PCKD: Polycystic Kidney Disease

Diagnosis	Mild (%)	Moderate (%)	Severe (%)	Chi-square value	p-value
PUV	4 (36.4%)	6 (54.5%)	1 (9.1%)	12.635	0.06
VUR	5 (55.6%)	2 (22.2%)	2 (22.2%)		
PUJO	4 (57.1%)	2 (28.6%)	1 (14.3%)		
MCDK	4 (66.7%)	0 (0.0%)	2 (33.3%)		
ERP	3 (60.0%)	2 (40.0%)	0 (0.0%)		
PCKD	3 (60.0%)	2 (40.0%)	0 (0.0%)		
Transient	3 (60.0%)	0 (0.0%)	2 (40.0%)		
Double Moiety	1 (50.0%)	1 (50.0%)	0 (0.0%)		

Conservative management with regular follow-up was the predominant approach, implemented in 42 (84.0%) participants, while surgical intervention was required in only 8 (16.0%), as depicted in Table 6.

**Table 6:** Management Approach for Antenatal Hydronephrosis Values are expressed as frequency and percentage.

Management	N	%
Follow-up (Conservative)	42	84.0%
Surgery	8	16.0%

Analysis revealed a strong correlation between the severity of hydronephrosis and the management approach. The majority of participants with mild [26 (96.2%)] and moderate [14 (86.6%)] hydronephrosis were successfully managed conservatively, while a substantial proportion of those with severe hydronephrosis [5 (62.5%)] required surgical intervention, as depicted in Table 7. The highly significant chi-square value (χ² = 15.99, p = 0.00034) confirms the observed pattern, indicating that the likelihood of requiring surgical intervention increases with the severity of hydronephrosis. This association underscores the importance of severity as a key determinant in management decisions, providing valuable prognostic information for clinical decision-making and parental counselling regarding the expected treatment approach.

**Table 7:** Correlation Between Hydronephrosis Severity and Management Approach Values are expressed as frequency and percentage. The p-value is by chi-square test, and <0.05 is considered statistically significant.

Management	Mild (%)	Moderate (%)	Severe (%)	Total (%)	Chi-square value	p-value
Conservative	26 (96.2%)	14 (86.6%)	3 (37.5%)	42 (84%)	15.9	<0.001
Surgical	1 (3.8%)	2 (13.4%)	5 (62.5%)	8 (16%)		
Total	27 (54%)	15 (30%)	8 (16%)	50 (100%)		

## Discussion

This prospective study evaluated the clinical features, etiological distribution, and management outcomes in 50 neonates and infants diagnosed with antenatal hydronephrosis (ANH). We analyzed multiple clinical variables, including gender, gestational age at detection, laterality, severity, and postnatal diagnoses. Our findings both corroborate existing literature and offer additional insights into diagnostic patterns and treatment outcomes.

A notable male predominance of 36 (72%) was observed in our cohort, aligning with prior studies that identified a higher incidence of urological anomalies in male infants. Previous studies have reported male prevalence rates exceeding 60% in ANH cohorts, supporting the male predominance observed in our study. Some reports have documented male predominance approaching 75%, further aligning with our findings [10,11]. The anatomical structure of the male urethra, particularly its susceptibility to posterior urethral valves (PUV) - a condition exclusive to males - likely contributes to this disparity [12].

Regarding the timing of diagnosis, 26 (52%) of ANH cases in our study were identified between 20-24 weeks gestation, coinciding with standard mid-trimester anomaly scans. This reflects the pivotal role of second-trimester ultrasonography in the early detection of urinary tract anomalies, as supported by earlier literature [13]. A smaller subset was diagnosed after 29 weeks, likely through surveillance ultrasounds or follow-up imaging for earlier findings. Continued monitoring beyond the initial screening window has been similarly emphasized [14].

Our laterality analysis revealed an equal prevalence of right-sided (36%) and bilateral hydronephrosis (36%), with a slightly lower frequency on the left side (28%). Earlier studies have described left-sided involvement in a similar proportion of cases, while others have reported left-sided hydronephrosis in roughly 40-45% of cases [15,16]. Variations in laterality patterns may be influenced by demographics, sample size, or ultrasound interpretation standards. Importantly, bilateral hydronephrosis is consistently associated with a higher risk of clinically significant pathology requiring intervention [4].

In terms of severity, mild hydronephrosis was most prevalent (54%), consistent with prior findings [9,17,18]. This severity distribution has important implications for postnatal care planning, as mild cases often resolve spontaneously, whereas severe cases more frequently necessitate intervention.

Postnatal imaging was guided by severity and clinical suspicion. All infants underwent ultrasonography, and selected patients underwent additional imaging with micturating cystourethrogram (48%) or radionuclide scans (44%). Comparable imaging utilisation patterns have been reported in other neonatal hydronephrosis studies [3,19,20]. This tiered approach aims to balance diagnostic accuracy with avoidance of unnecessary invasive testing, particularly in mild cases [21].

In our cohort, posterior urethral valves (PUV) were the most common postnatal diagnosis (22%), followed by vesicoureteral reflux (18%) and pelviureteric junction obstruction (14%). Previous studies have similarly identified PUJO and VUR as among the most common etiologies. Some reports have described transient hydronephrosis as the leading cause in certain populations, depending on follow-up duration and diagnostic criteria [22,23]. Multicystic dysplastic kidney (MCDK) occurred in 12% of cases, consistent with published rates [11].

Although we observed trends linking severity with specific diagnoses - such as moderate hydronephrosis in most PUV cases - these associations did not reach statistical significance (p = 0.06). Larger cohorts have demonstrated correlations between hydronephrosis severity and underlying etiology, but our limited sample size may have reduced statistical power [24,25].

Radionuclide scans demonstrated normal function in 50%, obstruction in 30%, and VUR-related findings in 20%. Similar patterns of radionuclide scan findings have been described in previous literature and support the view that many ANH cases are non-obstructive or transient [26,27].

A majority of 84% of our patients were managed conservatively, with only 16% requiring surgical intervention. This aligns with prior studies advocating non-operative management in most cases [1,2,4,23,28]. Other studies have similarly emphasized that a substantial proportion of ANH cases resolve without intervention, reinforcing the value of structured follow-up and judicious use of investigations [18].

The most significant finding in our study was the strong correlation between the severity of hydronephrosis and the management approach. Among the eight patients who underwent surgery, five (62.5%) had severe hydronephrosis, two (13.4%) had moderate, and one (3.8%) had mild, while conservative management predominated in mild and moderate cases (p < 0.001). This indicates that the likelihood of surgical intervention increases with increasing severity [29].

Several limitations warrant consideration, including the relatively small sample size, single-centre design, and limited follow-up duration, which may have impacted long-term outcome assessment and generalizability.

## Conclusions

This study highlights key clinical patterns in antenatal hydronephrosis, showing male predominance and diagnosis mainly during the second-trimester anomaly scan. Mild cases were most common, with PUV, VUR, and PUJO as leading causes. Severity strongly correlated with management - 62.5% of severe cases needed surgery, while most mild to moderate cases were managed conservatively. These findings reinforce the role of structured postnatal evaluation and selective imaging in optimizing care.
